# The Predictive Significance of Prognostic Nutritional Index and Serum Albumin/Globulin Ratio on the Overall Survival of Penile Cancer Patients Undergoing Penectomy

**DOI:** 10.3390/curroncol29100596

**Published:** 2022-10-11

**Authors:** Wei-Jie Song, Ni-Chujie Li, Jun Gao, Zhi-Peng Xu, Jian-Ye Liu, Zhi Long, Le-Ye He

**Affiliations:** 1 Department of Urology, Central South University, The Third Xiangya Hospital, Changsha 410013, China; 2 Sexual Health Research Center, Central South University, The Third Xiangya Hospital, Changsha 410013, China

**Keywords:** prognostic nutritional index, albumin/globulin ratio, penile cancer, penectomy, overall survival, prognostic factors

## Abstract

**Objective:** To assess the value of using the prognostic nutritional index (PNI) and serum albumin/globulin ratio (AGR) in predicting the overall survival (OS) of patients with penile cancer (PC) undergoing penectomy. **Materials and methods:** A retrospective analysis of 123 patients who were admitted to our hospital due to PC from April 2010 to September 2021 and who underwent penectomy were included in the study. The optimal cut-off value of the PNI and AGR was determined by receiver operating characteristic curve analysis. Kaplan–Meier analysis and the Cox proportional hazard model were used to evaluate the correlation between the PNI, AGR, and OS in patients with PC. **Results:** A total of 16 of the 123 patients died during the follow-up period, and the median follow-up time was 58.0 months. The best cut-off values of the PNI and AGR were set to 49.03 (95% confidence interval 0.705–0.888, Youden index = 0.517, sensitivity = 57.9%, specificity = 93.7%, *p* < 0.001) and 1.28 (95% confidence interval 0.610–0.860, Youden index = 0.404, sensitivity = 84.1%, specificity = 56.2%, *p* = 0.003). The Kaplan–Meier analysis showed that the OS of the patients in the high PNI group and the high AGR group was significantly higher than that of the patients in the low PNI group and the low AGR group (*p* < 0.001). The univariable analysis showed that the aCCI, the clinical N stage, the pathological stage, and the PNI, AGR, SII, and PLR are all predictors of OS in patients with PC (*p* < 0.05). The multivariable analysis showed that the PNI (risk rate [HR] = 0.091; 95% CI: 0.010–0.853; *p* = 0.036) and the AGR (risk rate [HR] = 0.171; 95% CI: 0.043–0.680; *p* = 0.012) are independent prognostic factors for predicting OS in patients with PC undergoing penectomy. **Conclusions:** Both the PNI score and the serum AGR are independent prognostic factors for predicting OS in patients with PC undergoing penectomy.

## 1. Introduction

Penile cancer (PC) is a malignant tumor that originates from the head of the penis, the coronal sulcus, the inner plate mucosa of the foreskin, or the skin of the penis. Due to demographic differences in many countries, including ethnic groups, religions, and sanitary conditions, the incidence of PC in different parts of the world varies widely. The incidence of PC has been reported to be as high as 19:100,000 men in some underdeveloped Asian, African, and Latin American countries, while the incidence in developed regions, such as Europe and the United States, is only about 0.1–0.9:100,000 men [1]. PC has become a rare men’s malignancy in China, accompanying the improvement of China’s medical and health level. Although both patients and doctors do not hope to perform a penectomy for PC patients, due to the fact that the penis is a sign of maleness, partial or radical penectomy is still the best choice for the treatment of late stage or deep infiltrating tumors in the head of the penis [2]. The prognostic assessments of PC are mainly based on histopathology and clinical staging, and there is a lack of effective clinical biomarkers [3]. Therefore, identifying novel clinical prognostic parameters to assist in predicting the clinical outcome of patients with PC after penectomy is critical.

Some commonly used inflammatory nutritional biomarkers have been found, such as the albumin/globulin ratio (AGR), the prognostic nutritional index (PNI), the systemic immune-inflammation index (SII), the neutrophil–lymphocyte ratio (NLR), and the platelet–lymphocyte ratio (PLR), which can predict the prognosis of cancer patients [4,5,6]. To the best of our knowledge, there are no relevant studies evaluating the inflammatory nutritional biomarkers as predictive markers for the prognosis of patients receiving penectomy. Therefore, we conducted this study.

## 2. Patients and Methods

### 2.1. Patients

We retrospectively analyzed the clinical data of 123 patients who underwent penectomy (partial/total penectomy) in our hospital from April 2010 to September 2021. Our study was approved by the Institutional Review Board (IRB) of The Third Xiangya Hospital of Central South University (Grant No. 21154). All the patients were diagnosed with PC through clinical laboratory testing, imaging examinations, and the biopsy of penile masses before surgery. The exclusion criteria included: (1) incomplete clinical data or follow-up data; (2) patients suffering from diseases that affect systemic nutrition or immune status, such as autoimmune diseases; (3) patients with blood infections or blood diseases, such as leukemia; (4) patients taking any immunosuppressive drugs or any nutritional supplements before surgery; and (5) patients with other tumor diseases or with lymph node or distant metastasis before surgery.

### 2.2. Data Collection

All the patients included in our study underwent routine blood work and liver and kidney function assessments one week before surgery. The definitions of the prognostic nutritional index (PNI), the albumin/globulin ratio (AGR), the systemic immune-inflammation index (SII), the neutrophil–lymphocyte ratio (NLR), and the platelet–lymphocyte ratio (PLR) were shown as follows: PNI = 1 × serum albumin (g/L) + 5 × lymphocyte count (109/L); AGR = serum albumin/(total serum protein-serum albumin); SII = platelet × neutrophil/lymphocyte count; NLR = neutrophil/lymphocyte count; and PLR = platelet/lymphocyte count. The collected clinical pathological data included the age of the patient, the scope of penile resection, necrosis, clinical T and N grades, and pathological types. The clinical staging of the tumors was determined according to the Tumor, Lymph Node, and Metastasis (TNM) staging system of the Joint Committee on Cancer of the United States (7th Edition) [7]. The data presented in this study are available in the Supplementary Material.

### 2.3. Follow-Up

Follow-up information was obtained by searching for outpatient review information and telephone follow-up with the patients. The follow-up time was defined as the date of diagnosis of PC and penectomy to 31 September 2021. Follow-up occurred every four months for the first two years after surgery and every six months from the third year after surgery onward. The follow-up included a physical examination, functional assessment, blood biochemical test, and a thorough examination of patients to determine any possibility of recurrence or metastasis.

### 2.4. Statistical Analysis

All the data were processed using SPSS v25.0 statistical software. The highest value of the Youden index was calculated using the receiver operating characteristic (ROC) curve as the best cut-off value of the PNI, AGR, SII, PLR, and NLR and grouped separately. When the measurement data conformed to a normal distribution, mean ± standard deviation was used. The measurement data that did not conform to a normal distribution were expressed as the median (range). The Kaplan–Meier survival analysis and log-rank test were used to compare the effects of different groups on overall survival (OS) in patients with PC undergoing penectomy. The variables with significant differences in the univariable analysis (*p* < 0.05) were included in the Cox proportional hazards regression model for multivariable survival analysis, and the hazard ratio (HR) and 95% confidence interval (CI) were estimated. Differences with a *p* < 0.05 were considered to be statistically significant.

## 3. Results

### 3.1. Patients’ Information

A total of 123 patients with PC who underwent penectomy were included in this study, of which 101 (82.1%) underwent partial penectomy and 22 (17.9%) underwent total penectomy. The median follow-up time of the 123 patients was 58 months. The median follow-up time of the 107 patients who survived was 61 months. The age of the patients ranged from 29 to 90 years old, with a median age of 59 years. The preoperative comorbidities of the patients were quantified by the age-adjusted Charlson comorbidity index (aCCI) [8]. The median value of the aCCI was 3.00. There were 94 cases (76.4%) with T < 2 in the clinical stage (Tis 12 cases, Ta 45 cases, and T1 37 cases) and 29 cases (23.6%) with T ≥ 2 (T2 27 cases, T3 1 case, and T4 1 case). In the clinical stage, there were 115 cases (93.5%) with *n* = 0 and 8 cases (6.5%) with *n* > 0 (They are the inguinal lymphadenopathy found during clinical palpation, which should be inflammatory enlargement.). Pathological classification showed that there were 12 cases (9.8%) of carcinoma in situ and 111 cases (90.2%) of squamous cell carcinoma. The median values of the preoperative PNI, AGR, SII, PLR, and NLR were 49.30, 1.51, 464.53, 118.52, and 2.33, respectively. Table 1 summarizes the clinicopathological characteristics of the patients in our study.

### 3.2. The Best Cut-Off Values of PNI, AGR, SII, and PLR before Treatment

**Figure 1:**
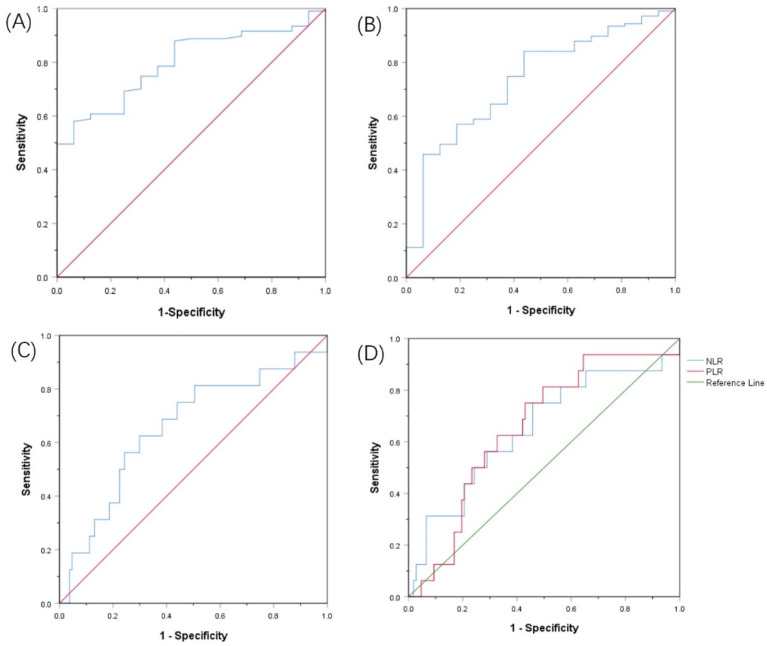
Receiver operating characteristic curves for pretreatment: (**A**) PNI, (**B**) AGR, (**C**) SII, (**D**) RLR and NLR based on OS.

The cut-off values for the PNI, AGR, SII, and PLR were determined by the ROC curve, and the optimal cut-off values were set at 49.03, 1.28, 636.99, and 121.95, respectively. The area under the curve (AUC) of the PNI was 0.797 (95% CI: 0.705–0.888, Youden index = 0.517, sensitivity = 57.9%, specificity = 93.7%, *p* < 0.001). The AUC of the AGR was 0.735 (95% Cl: 0.610–0.860, Youden index = 0.404, sensitivity = 84.1%, specificity = 56.2%, *p* = 0.003). The AUC of the SII was 0.657 (95% Cl: 0.505–0.808, Youden index = 0.326, sensitivity = 62.5%, specificity = 70.1%, *p* = 0.044). The AUC of the PLR was 0.654 (95% Cl: 0.521–0.787, Youden index = 0.320, sensitivity = 75.0%, specificity = 57.0%, *p* = 0.047) (Figure 1).

### 3.3. Comparison of Clinical Data of Patients in Different Groups

The optimal cut-off values of the PNI, AGR, SII, and PLR were used as the standard, and the patients below the cut-off value were defined as the low-level group, while those higher than or equal to the cut-off value were defined as the high-level group. Because the maximum area under the curve when NLR = 2.34 is 0.648 (95%Cl 0.493–0.803, Youden index = 0.292, sensitivity = 75.0%, specificity = 54.2%, *p* = 0.057), *p* > 0.05 has no statistical significance. Therefore, it is not used as a standard for grouping. The clinical N stage of the patients in the low AGR group was worse than that in the high AGR group (*p* = 0.039, Table 2). In addition, compared with the low SII group, the high SII group retained less penile tissue. (*p* = 0.027, Table 2).

### 3.4. Kaplan–Meier Survival Analysis

**Figure 2:**
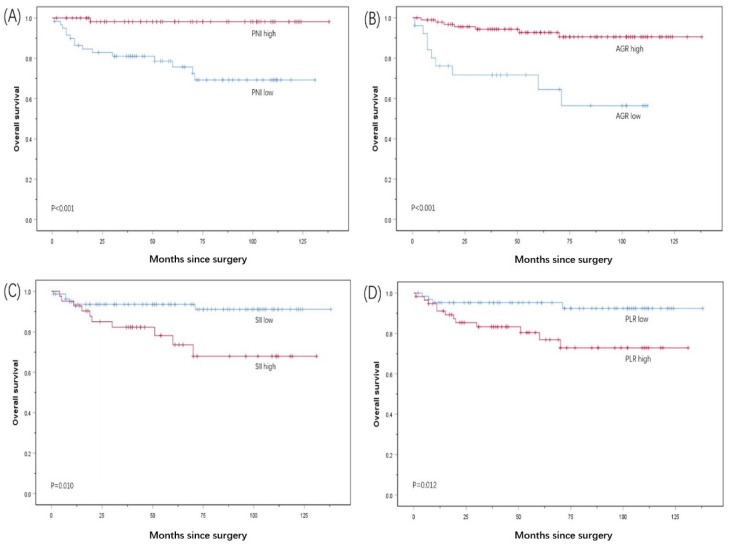
Kaplan–Meier survival curves for OS according to (**A**) PNI, (**B**) AGR, (**C**) Sll, and (**D**) PLR in PC patients.

The Kaplan–Meier survival analysis showed that the median OS of the patients with a PNI < 49.03 was 100.4 months, which was lower than the median OS of the patients with a PNI ≥ 49.03 of 135.8 months (*p* < 0.001). The median OS of the patients with an AGR ≥ 1.28 before surgery was 128.2 months, which was significantly higher than the median OS of 75.7 months for the patients with an AGR <1.28 (*p* < 0.001). The median OS of the patients with an SII ≥ 636.99 before surgery was 10.5 months, which was significantly lower than the median OS of the patients with an SII < 636.99 of 128.0 months (*p* = 0.010). The median OS of the patients with a PLR ≥ 121.95 before surgery was 104.2 months, which was significantly lower than the median OS of the patients with a PLR < 121.95 of 129.9 months (*p* = 0.012) (Figure 2).

### 3.5. Univariable and Multivariable Analysis

**Figure 3:**
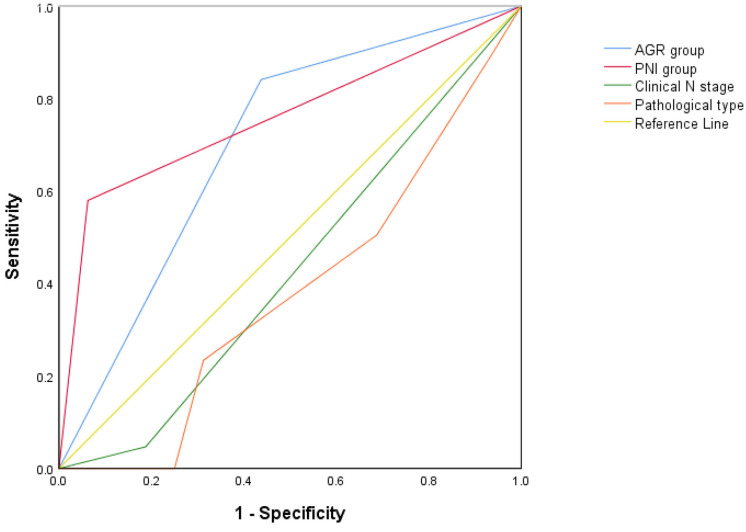
Receiver operating characteristic curves for all the independent factors of multivariable analysis.

The results of the univariable analysis showed that the age-adjusted Charlson comorbidity index (aCCI), clinical N staging, pathological classification, and the preoperative PNI, AGR, SII, PLR, and OS of patients with PC were statistically correlated (all *p* < 0.05, Table 3). The multivariable analysis revealed that the clinical N stage (*p* = 0.033; Table 3), pathological type (*p* = 0.045; Table 3), preoperative PNI (risk rate [HR] = 0.091; 95% CI: 0.010–0.853; *p* = 0.036; Table 3), and AGR (risk rate [HR] = 0.171; 95% CI: 0.043–0.680; *p* = 0.012; Table 3) were all independent prognostic factors. Thus, we plotted new ROC curves combining all the factors that were statistically different in the multivariate analysis (Figure 3). The AUC of the PNI group was 0.758 (95% CI: 0.655–0.862, *p* = 0.001). The AUC of the AGR group was 0.702 (95% CI: 0.549–0.854, *p* = 0.009). The AUC of the clinical N stage was 0.430 (95% CI: 0.267–0.592, *p* = 0.365). The AUC of the pathological type was 0.380 (95% CI: 0.223–0.537, *p* = 0.123). *p* > 0.05 has no statistical significance. Therefore, we finally found that the PNI and AGR are better independent prognostic factors for OS in patients with PC treated with penectomy.

## 4. Discussion

PC is a relatively rare men’s malignant tumor, which can seriously affect the quality of life of men. In the process of the diagnosis and treatment of penile cancer, clinicians need to choose scientific and effective diagnosis and treatment methods, which not only consider the overall survival rate of patients, but also avoid overtreatment, such as unnecessary penectomy and inguinal lymphadenectomy. There is still a lack of specific prognostic biomarkers for PC; so, it is very important to explore reliable clinical markers for the diagnosis and treatment of PC. In this study, we evaluated the prognostic value of the PNI score and serum AGR in predicting OS in patients with PC. Both the PNI score and the serum AGR can reflect the systemic nutrition and inflammation status of tumor patients, and both are non-invasive biomarkers.

The latest research shows that systemic malnutrition and inflammation are closely related to the poor prognosis of various malignant tumors [9,10]. Thus far, a variety of proteins have been found in human plasma, among which albumin accounts for more than half [11]. Serum albumin (ALB), a central water-soluble protein produced by the liver, maintains the osmotic pressure of the capillaries and eliminates free radicals in the blood. It is also commonly used in clinical practice to assess the nutritional status of patients [12]. Malnutrition can lead to delayed postoperative healing and decreased collagen synthesis, thereby damaging the body’s immune system [13]. At the same time, malnutrition can lead to increased mortality after surgery and can reduce the resistance of patients to malignant tumors [14]. A study on ALB and cancer found that the serum ALB level of cancer subjects was significantly lower than that of non-cancer subjects [15]. ALB is also closely related to the prognosis of a variety of cancers and can be used as a predictor of the recurrence, metastasis, and death of a variety of malignant tumors [16,17,18]. In a study of a physical examination crowd, the correlation between low levels of ALB and tumor morbidity and mortality was also found [19]. In addition to being a nutritional marker, ALB is also an inflammatory response index [20]. Various pro-inflammatory cytokines can regulate the synthesis of albumin in the hepatocytes as part of the tumor inflammatory response and, at the same time, can promote tumor growth and metastasis, destroy the host’s immune response, and promote drug resistance [21]. Numerous studies have confirmed that markers of chronic inflammation play a key role in cancer recurrence, metastasis, and progression and are closely related to the OS of a variety of malignant tumors [22,23,24].

The PNI index is calculated from the ALB and lymphocyte count and can reflect the body’s nutritional and inflammatory state to a certain extent. Lymphocytes also play a very important role in the immune surveillance of malignant tumors. They can inhibit the proliferation and metastasis of malignant tumors by promoting the production of cytokines and the apoptosis of cytotoxic cells [25]. At present, researchers have studied the role of the PNI index in the prognosis of various malignant tumors. The total tumor recurrence rate and OS of patients in the higher PNI score group were higher than those in the lower PNI score group. The PNI index has been found to be an independent prognostic factor for a variety of malignant tumors, but none of these reports assessed the prognosis of PC [26,27,28].

Serum AGR is calculated by the ratio of ALB to serum globulin and can reflect the patient’s nutritional and inflammatory state. Serum globulin is calculated by subtracting the ALB from the total serum protein. Therefore, it contains a variety of pro-inflammatory proteins, such as C-reactive protein (CRP), immunoglobulin (Igs), and complement components. Studies have shown that elevated levels of CRP before surgery can lead to poor cancer prognosis [29]. Studies of patients with lung cancer have also found that a higher serum globulin level was associated with a lower survival rate [30]. Additionally, studies of patients with colorectal cancer have found that high complement and IgA levels are also poor prognosis indicators [31]. Serum AGR has also been confirmed as an independent prognostic factor for a variety of malignant tumors [16,32,33].

Squamous cell carcinoma is the most common pathological type of PC, accounting for about 95% of patients with PC. The pathological type of all the patients in our study was squamous cell carcinoma. Recently, some researchers found that the PNI index and serum AGR level are significantly related to the OS of patients with esophageal cancer [34,35], oral cancer [36,37], and laryngeal cancer [38,39]. The researchers conducted a multivariate analysis of the PNI index and serum AGR level with the patients’ comorbidities, age, and other factors and found that both the PNI and the AGR were independent predictors of postoperative OS, which is consistent with our research conclusions.

## 5. Conclusions

In this study, we found that both the PNI score and the serum AGR can be used as independent prognostic factors for predicting OS in patients with PC undergoing penectomy. Higher PNI scores and higher AGR levels are associated with longer OS for patients with PC. We confirmed the importance of preoperative nutritional support and inflammation control for the prognosis of patients with PC undergoing penectomy. Additionally, we found that poor clinical staging and higher levels of PLR and SII are related to the poor prognosis of patients with PC. The PLR and SII are also commonly used clinical nutrition and inflammation markers, once again corroborating the results of this study. However, there are limitations to our study. On the one hand, our retrospective study was with a small sample size of 123 patients. Therefore, our results require further confirmation by a multi-institutional, prospective study with a large number of patients to provide a better conclusion. On the other hand, our research lacks the measurement of specific inflammatory markers such as CRP and cytokine levels. Therefore, whether the preoperative PNI score and AGR are valid as prognosis indicators for patients with PC requires further investigation and verification.

## Figures and Tables

**Table 1:** Characteristics of the 123 patients.

Variables	Total *n* = 123	%
Age, y		
˂60	64	52
≥60	59	48
aCCI median (IQR)	3.00 (3.00–4.00)	
ZPS		
0	120	97.6
1	3	2.4
Penis removal range		
Part	101	82.1
Entire	22	17.9
Necrosis		
No	113	91.9
Yes	10	8.1
T stage		
T < 2	94	76.4
T ≥ 2	29	23.6
Clinical *n* stage		
*n* = 0	115	93.5
*n* > 0	8	6.5
Pathological type		
Carcinoma in situ	12	9.8
Squamous cell carcinoma	111	90.2
Verrucous	46	37.4
Highly differentiated	35	28.5
Moderately differentiated	26	21.1
Poorly differentiated	4	3.2
PNI median (IQR)	49.30 (45.20–52.90)	
AGR median (IQR)	1.51 (1.32–1.72)	
SII median (IQR)	464.53 (358.50–757.33)	
PLR median (IQR)	118.52 (92.14–149.19)	
NLR median (IQR)	2.33 (1.78–3.20)	
Follow-up time (months) median (IQR)	58.00 (27.00–97.00)	

IQR: interquartile range; aCCI: age-adjusted Charlson comorbidity index; ZPS: performance status Zubrod-ECOG-WHO.

**Table 2:** Comparison of different PNI/AGR/SII/PLR groups.

		PNI			AGR			SII			PLR	
Variables	Low	High	*p*	Low	High	*p*	Low	High	*p*	Low	High	*p*
	(60)	(63)		(26)	(97)		(81)	(42)		(65)	(58)	
Age, y			0.061			0.501			0.279			0.671
˂60	26	38		12	52		45	19		35	29	
≥60	34	25		14	45		36	23		30	29	
ZPS			0.532			0.602			0.231			0.064
0	58	62		25	95		80	40		65	55	
1	2	1		1	2		1	2		0	3	
Penis removal range			0.125			0.178			**0.027**			0.089
Part	46	55		19	82		71	30		57	44	
Entire	14	8		7	15		10	12		8	14	
Necrosis			0.461			0.476			0.272			0.398
No	54	59		23	90		76	37		61	52	
Yes	6	4		3	7		5	5		4	6	
T stage			0.565			0.966			0.843			0.732
T < 2	45	49		20	74		62	32		50	44	
T ≥ 2	15	14		6	23		19	10		15	14	
Clinical N stage			0.943			**0.039**			0.330			0.104
*n* = 0	56	59		22	93		77	38		63	52	
*n* > 0	4	4		4	4		4	4		2	6	
Histological type			0.751			0.396			0.303			0.267
Carcinoma in situ	6	6		3	9		8	4		6	6	
Squamous cell carcinoma	54	57		23	88		73	38		59	52	
Verrucous	24	22		7	39		32	14		26	20	
Highly differentiated	12	23		8	27		24	11		22	13	
Moderately differentiated	14	12		7	19		16	10		10	16	
Poorly differentiated	4	0		1	3		1	3		1	3	

ZPS: Performance Status Zubrod-ECOG-WHO. Bold values indicate the statistically significant *p* values (*p* ≤ 0.05).

**Table 3:** Univariate and multivariate analysis of factors.

Variables	Univariate Analysis			Multivariate Analysis		
	HR	95% CI	*p* Value	HR	95% CI	*p* Value
Age, y			0.077			
˂60	1					
≥60	2.596	0.901–7.481				
aCCI	2.676	1.544–4.637	**<0.001**	1.327	0.623–2.823	0.463
ZPS			0.082			
0	1					
1	6.276	0.792–49.751				
Penis removal range			0.107			
Part	1					
Entire	2.393	0.828–6.913				
Necrosis			0.226			
No	1					
Yes	2.543	0.561–11.524				
T stage			0.324			
T < 2	1					
T ≥ 2	1.703	0.591–4.901				
Clinical *n* stage			**0.011**			**0.033**
*n* = 0	1			1		
*n* > 0	5.243	1.454–18.898		7.553	1.178–48.415	
Pathological type			**<0.001**			**0.045**
Carcinoma in situ	1			1		
Squamous cell carcinoma						
Verrucous	0.934	0.104–8.356		1.424	0.149–13.635	
Highly differentiated	1.994	0.240–16.581		3.133	0.366–26.786	
Moderately differentiated	0.449	0.028–7.184		0.321	0.013–7.954	
Poorly differentiated	32.025	3.418–300.096		20.158	1.571–258.591	
NLR	1.077	0.950–1.221	0.243			
PNI			**0.007**			**0.036**
Low	1			1		
High	0.061	0.008–0.462		0.091	0.010–0.853	
AGR			**<0.001**			**0.012**
Low	1			1		
High	0.160	0.059–0.429		0.171	0.043–0.680	
SII			**0.017**			0.146
Low	1			1		
High	3.457	1.251–9.551		2.512	0.725–8.703	
PLR			**0.022**			0.321
Low	1			1		
High	3.772	1.212–11.737		0.449	0.093–2.180	

aCCI: age-adjusted Charlson comorbidity index; ZPS: performance status Zubrod-ECOG-WHO; IQR: interquartile range. Bold values indicate the statistically significant *p* values (*p* ≤ 0.05).

## Data Availability

The data presented in this study are available in the Supplementary Material.

## Supplementary Materials

The following supporting information can be downloaded at: https://www.mdpi.com/article/10.3390/curroncol29100596/s1, the original data.
